# The impact of the COVID-19 pandemic on the mental health of nursing professionals in the state of São Paulo

**DOI:** 10.1192/j.eurpsy.2024.1079

**Published:** 2024-08-27

**Authors:** M. C. V. R. De Oliveira, E. Ribeiro dos Santos, E. C. Araujo

**Affiliations:** ^1^ Medical College, UNOESTE, PRESIDENTE PRUDENTE; ^2^ Regional Nursing Council of the State of São Paulo Brazil, COREN-SP; ^3^CORENSP, SÃO PAULO, Brazil

## Abstract

**Introduction:**

This is a descriptive cross-sectional clinical study with professionals from the Nursing Team (Nursing Assistant, Nursing Technician and Nurse).

**Objectives:**

To assess the psychological impact of the Covid-19 pandemic on nursing staff professionals.

**Methods:**

A descriptive, quantitative, cross-sectional study will be applied to a structured interview aimed at collecting sociodemographic and occupational data, Mental Health Scales evaluating professional exhaustion - Oldenburg Burnout Inventory and Beck’s Anxiety Rating Scale to assess the state of anxiety.

**Results:**

About 13,587 nursing professionals were interviewed, including nurses, technicians and nursing assistants. They were evidenced through the behavior indexes related to insomnia, the desire to cry and appetite variation may be related to the long working hours, the fear of contamination and the consequent absence from work, as well as the fear of getting sick may be related to the fact that the professional stops being a caregiver and starts to be cared for.

**Conclusions:**

the study denotes the importance and need for interventions to promote and prevent mental well-being in health professionals exposed to COVID-19, these need to be implemented immediately, for nursing professionals, as they are on the front line, demanding attention Special. In this sense, the Nursing Council of the State of São Paulo created and implemented some bills such as the Obligation of Rest Rooms in Health Units, the Cuidando de Quem Cuida Program and the Yellow September Campaign in Allusion to actions for Nursing professionals for the prevention and promotion in mental health category.

**Disclosure of Interest:**

None Declared

